# Supplementary material for: Clomiphene Citrate Shows Effective and Sustained Antimicrobial Activity against Mycobacterium abscessus
Source: Int J Mol Sci. 2021 Oct 13;22(20):11029. doi: 10.3390/ijms222011029 (PMC8537717; doi:10.3390/ijms222011029)
Supplement: Supplementary file 1 [file ijms-22-11029-s001.zip › ijms-1391470-supplementary.pptx]

## Slide 1
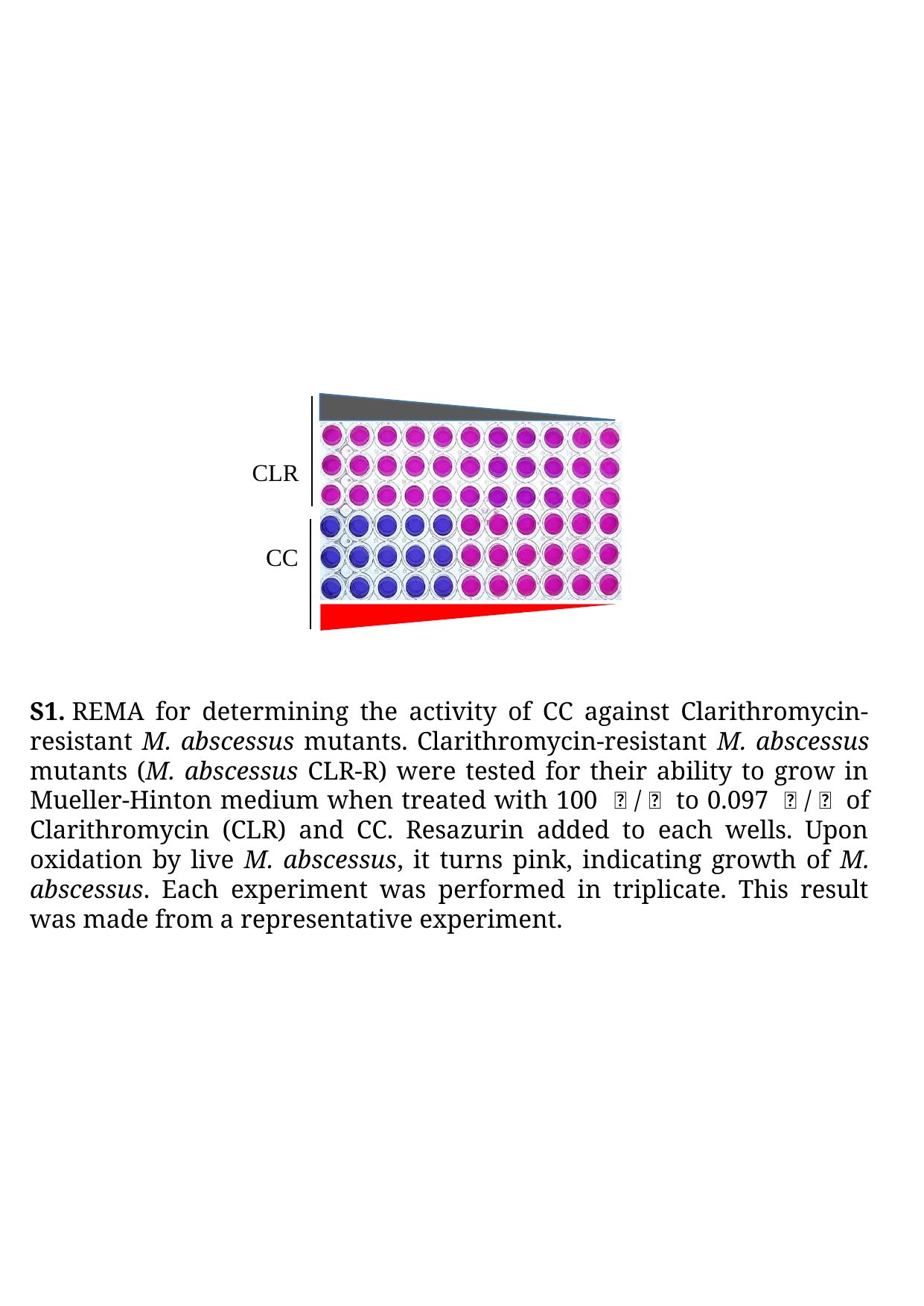

CLR
CC
S1. REMA for determining the activity of CC against Clarithromycin-resistant M. abscessus mutants. Clarithromycin-resistant M. abscessus mutants (M. abscessus CLR-R) were tested for their ability to grow in Mueller-Hinton medium when treated with 100 ㎍/㎖ to 0.097 ㎍/㎖ of Clarithromycin (CLR) and CC. Resazurin added to each wells. Upon oxidation by live M. abscessus, it turns pink, indicating growth of M. abscessus. Each experiment was performed in triplicate. This result was made from a representative experiment.

## Slide 2
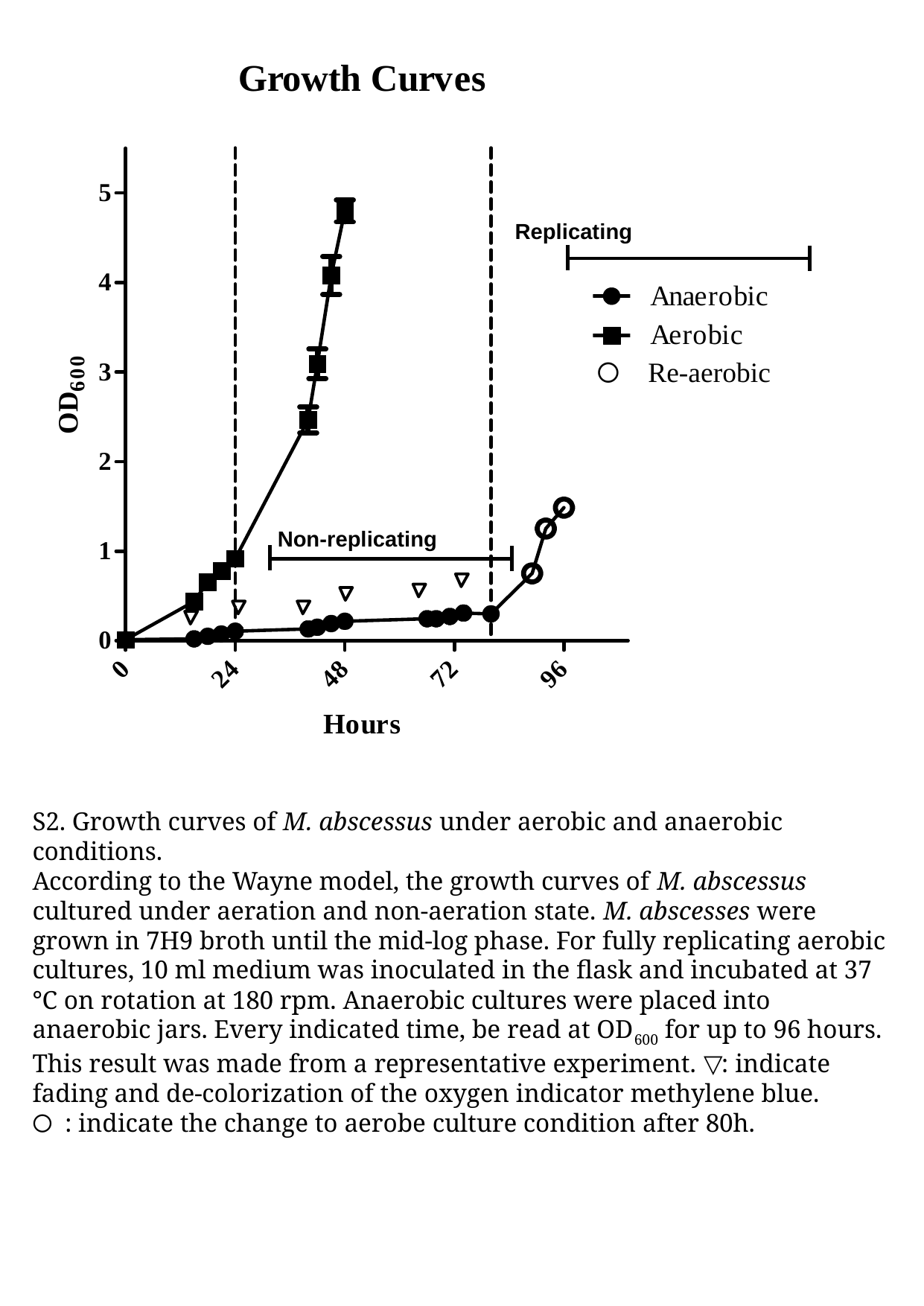

Replicating
〇 Re-aerobic
Non-replicating
S2. Growth curves of M. abscessus under aerobic and anaerobic conditions.
According to the Wayne model, the growth curves of M. abscessus cultured under aeration and non-aeration state. M. abscesses were grown in 7H9 broth until the mid-log phase. For fully replicating aerobic cultures, 10 ml medium was inoculated in the flask and incubated at 37 °C on rotation at 180 rpm. Anaerobic cultures were placed into anaerobic jars. Every indicated time, be read at OD600 for up to 96 hours. This result was made from a representative experiment. ▽: indicate fading and de-colorization of the oxygen indicator methylene blue.
〇 : indicate the change to aerobe culture condition after 80h.

## Slide 3
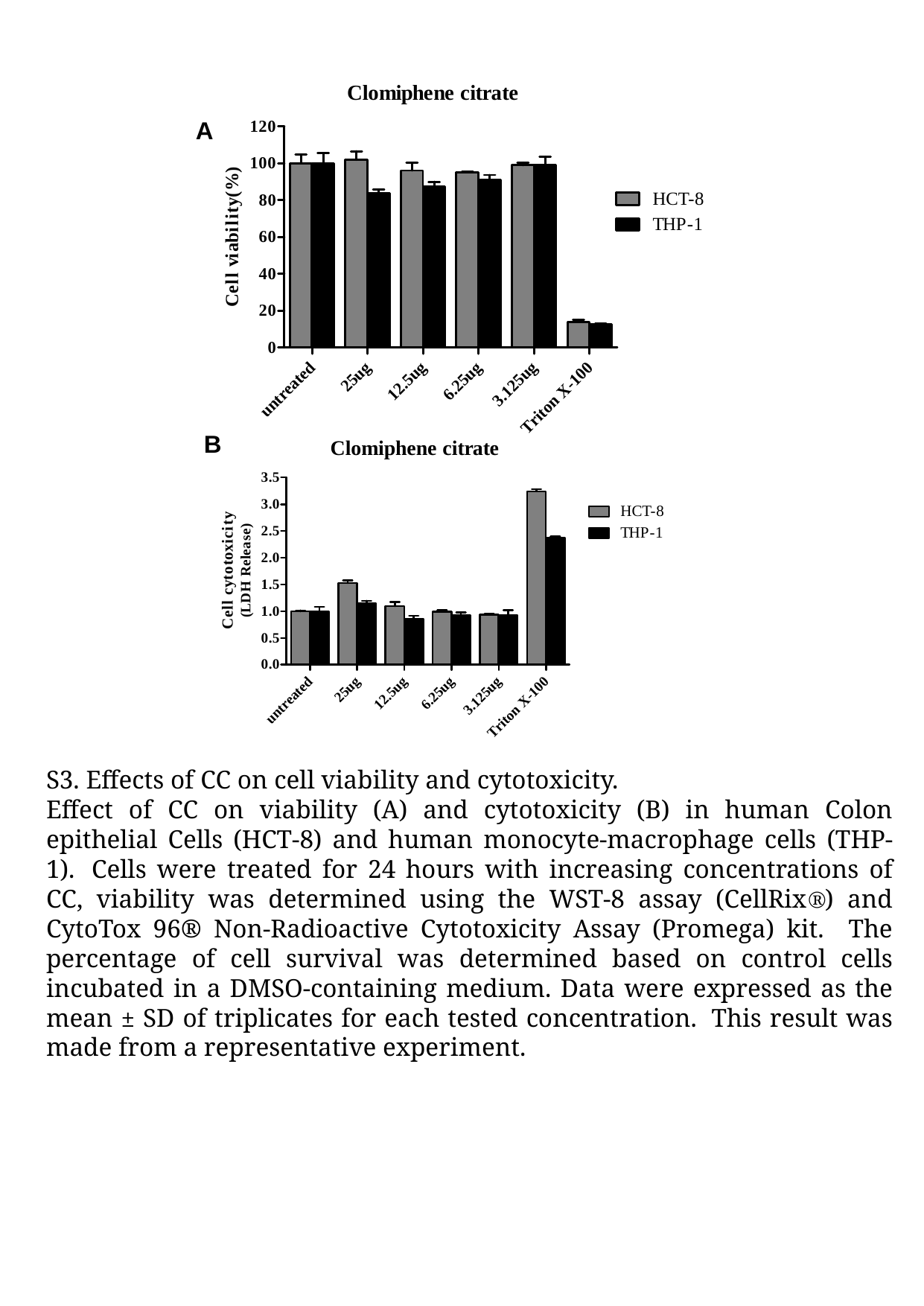

A
B
S3. Effects of CC on cell viability and cytotoxicity.
Effect of CC on viability (A) and cytotoxicity (B) in human Colon epithelial Cells (HCT-8) and human monocyte-macrophage cells (THP-1).  Cells were treated for 24 hours with increasing concentrations of CC, viability was determined using the WST-8 assay (CellRixⓇ) and CytoTox 96® Non-Radioactive Cytotoxicity Assay (Promega) kit. The percentage of cell survival was determined based on control cells incubated in a DMSO-containing medium. Data were expressed as the mean ± SD of triplicates for each tested concentration.  This result was made from a representative experiment.
